# ER-PM Contact Sites – SNARING Actors in Emerging Functions

**DOI:** 10.3389/fcell.2021.635518

**Published:** 2021-02-11

**Authors:** Bailey Hewlett, Neha Pratap Singh, Christian Vannier, Thierry Galli

**Affiliations:** ^1^INSERM U1266, Institut de Psychiatrie et Neurosciences de Paris, Université de Paris, Paris, France; ^2^GHU PARIS Psychiatrie and Neurosciences, Paris, France

**Keywords:** membrane contact sites, tethers, lipid transfer, SNAREs, neurons

## Abstract

The compartmentalisation achieved by confining cytoplasm into membrane-enclosed organelles in eukaryotic cells is essential for maintaining vital functions including ATP production, synthetic and degradative pathways. While intracellular organelles are highly specialised in these functions, the restricting membranes also impede exchange of molecules responsible for the synchronised and responsive cellular activities. The initial identification of contact sites between the ER and plasma membrane (PM) provided a potential candidate structure for communication between organelles without mixing by fusion. Over the past decades, research has revealed a far broader picture of the events. Membrane contact sites (MCSs) have been recognized as increasingly important actors in cell differentiation, plasticity and maintenance, and, upon dysfunction, responsible for pathological conditions such as cancer and neurodegenerative diseases. Present in multiple organelles and cell types, MCSs promote transport of lipids and Ca^2+^ homoeostasis, with a range of associated protein families. Interestingly, each MCS displays a unique molecular signature, adapted to organelle functions. This review will explore the literature describing the molecular components and interactions taking place at ER-PM contact sites, their functions, and implications in eukaryotic cells, particularly neurons, with emphasis on lipid transfer proteins and emerging function of SNAREs.

## Introduction

A distinctive property of eukaryotic cells is the compartmentaliasation of the cytoplasm by intracellular membranes. In addition to the plasma membrane (PM), phospholipid bilayers and monolayers form complex internal networks, defining individual organelles specialised for specific functions. While this is useful for separating incompatible biochemical reactions and restricting specific conditions such as low pH, and/or redox potential, compartmentalisation can impede the transfer of molecules which instead relies on vesicular trafficking (Helle et al., 2013; Scorrano et al., 2019). Recent studies however have shown that long known specialised regions of membrane in close proximity to each other, called membrane contact sites (MCSs), could also be involved in communication (Gallo et al., 2016; Kang et al., 2019; Bohnert, 2020). Early research identified closely apposed domains of the endoplasmic reticulum (ER) and mitochondria in hepatocytes (Bernhard and Rouiller, 1956) and of ER with T-tubule invaginations of the PM in muscle cells (Porter and Palade, 1957). However, the physiological significance of many MCSs was poorly understood. The first insights only arrived in the 1990s, with observations suggesting that phosphoserine-derivatives in the mitochondrial membranes are synthesised by the ER, and that contact between the organelles was involved in lipid transfer (Vance, 1990; Stefan et al., 2017). Since, MCSs have been identified in a range of organisms, cell types and between different organelles, the structure and function of many are still yet to be fully understood. MCSs are defined as domains of organelles in close apposition to each other, in general via biochemically defined interactions. They involve most organelles and can be homotypic (contacts between distal regions of an organelle or organelles of the same type) or heterotypic (between organelles of different nature) (Gatta and Levine, 2017; Scorrano et al., 2019). Due to its extensive and dynamic network throughout the cell (Nixon-Abell et al., 2016), the ER is engaged as a partner in almost all heterotypic connections and mitochondria have also been shown to form contacts with multiple organelles (Gatta and Levine, 2017).

The diversity of interactions observed suggests a variety of functions and processes that MCSs are involved in. For example, ER contacts with mitochondria are important in lipid transport and metabolism (Dimmer and Rapaport, 2017). Additionally, the ER can wrap around mitochondrial tubules and facilitate fission (Tilokani et al., 2018). Remarkably, numerous cellular processes depend on ER contacts beside organelle dynamics. ER-mediated contacts sites have been shown to facilitate Ca^2+^ homoeostasis (Wu et al., 2006) lipid transport (Tavassoli et al., 2013), modulate autophagic biogenesis (Nascimbeni et al., 2017), organelle morphology (Manford et al., 2012), and excitability in neurons and muscle cells (Ito et al., 2001; Sahu et al., 2019). Incidentally, several protein families of no obvious similarity have been identified at MCSs. Specific isoforms or even entirely separate families are allocated to MCSs depending on the membranes involved and the cell type, raising questions regarding their functionality and expression patterns. This review will summarize the literature describing the nature and molecular composition of ER-PM MCSs, presenting the roles of various populating protein families, emphasising MCSs in neurons and among related functions, the emerging role of SNAREs at MCSs.

## ER-PM Contact Sites in Neurons

### Main Features and Morphology

The ER forms an extensive network of tubules and cisternae throughout the cell and has a range of roles integral to cell survival and function including lipid synthesis, protein synthesis, and Ca^2+^ regulation. The network projects to all parts of the cell and is functionally connected to other organelles (Nixon-Abell et al., 2016). ER-mediated contact sites typically present the following characteristics: 1) the two membranes involved are tethered within 7 – 30 nm of each other, 2) despite this close proximity, membrane composition is maintained and there are no reports of fusion between organelles, and 3) specific proteins and lipids are enriched at MCSs, creating microdomains (Prinz, 2014; Eisenberg-Bord et al., 2016; Gallo et al., 2016). Depending on their type, MCSs can be transient or stable over time, according to their role in cellular structure and physiology, as noted in Orai–Stim (Helle et al., 2013) and Kv2.1 (Fox et al., 2015) induced tethering respectively. Importantly, the interactions at MCSs influence the function of either or both the participating organelles.

Endoplasmic reticulum-mediated MCSs also show specific characteristics. Studies in *Saccharomyces cerevisiae* indicate that tethering another membrane excludes ribosomes from the cytoplasmic face of the ER (Wolf et al., 2012) and controls membrane curvature, vital for maintaining membrane integrity (Collado et al., 2019). The ER MCSs with other organelles, including mitochondria and peroxisomes (Elbaz and Schuldiner, 2011), are some of the best characterized examples of MCSs and have been described in multiple cell types and organisms, mediating additional processes at each contact site.

Initial evidence for ER-PM MCSs in neurons and muscle cells came from observation by electron microscopy, identifying subsurface cisternae “closely opposed” to the PM, maintaining a separation of 5–8 nm between the membranes (Porter and Palade, 1957; Rosenbluth, 1962). The high concentration of proteins in the membrane at these sites, as observed by freeze-fracture techniques, opened the possibility that these junctions had functions related to cell excitability (Henkart et al., 1976). Much later, this hypothesis was reinforced in rat hippocampal neurons (Spacek and Harris, 1997) with subsequent characterisation of novel tethers such as Kv2.1 K^+^ channels (Fox et al., 2015; Kirmiz et al., 2018) along with several SNARE proteins (Petkovic et al., 2014). 3D reconstructions show that the percentage of cell body PM engaged in MCSs with the ER as approximately 12.5% in the *Nucleus accumbens*, but varies in other brain regions (Wu et al., 2017). In agreement with earlier studies, the authors also found that, while the ER extends throughout neuronal cells, ER-PM MCSs are far less frequent in dendrites, only at the periphery of the post-synaptic densities and not at all in spines (Spacek and Harris, 1997; Wu et al., 2017). In contrast, yeast ER MCSs can cover 25–40% of the PM while separation reports have varied between studies, averaging 33 nm (West et al., 2011) but also reported lower at 21 nm (Collado et al., 2019). Several studies have also distinguished the morphology of ER associated with the PM in various models (West et al., 2011; Manford et al., 2012; Nixon-Abell et al., 2016; Wu et al., 2017). While the ER network surrounds the nucleus to form the nuclear envelope (Helle et al., 2013), tubules extend throughout the cell into the periphery, referred to as cortical ER (cER). Here the ER presents highly variable morphology, with “flattened sheets” with lumen width >25 nm (in neurons) (Orci et al., 2009; Wu et al., 2017) separated by thin tubules (Fernández-Busnadiego et al., 2015; Wu et al., 2017; Collado et al., 2019). Consistent with the dynamic nature of the ER, the larger sheets observed can quickly assemble and disassemble, likely due to interactions with motor proteins on microtubules (Nixon-Abell et al., 2016), which are frequently found in alignment with the cER (Orci et al., 2009). Extending into dendrites, the ER predominantly forms tubules and is less frequently in contact with the PM (Wu et al., 2017). ER-PM MCSs in the axons and dendrites of neurons are smaller and less frequent than in the cell body, and while the ER can enter the necks of large spines, they do not seem to form contacts (Spacek and Harris, 1997; Wu et al., 2017).

### Molecular Composition of ER-PM MCSs

Membrane contact sites could be viewed as microenvironments where selected proteins, namely tethering proteins, first establish physical bridge(s) between membranes, then recruit partner proteins which perform contact specific functions (Helle et al., 2013). In MCSs, membrane-associated tethering proteins maintain two distinct membranes in close apposition, under conditions where no membrane fusion occurs. Tethering proteins are often ER integral membrane proteins able to directly bind to either PM lipids or soluble lipid-binding proteins (Prinz, 2014; Eisenberg-Bord et al., 2016). Alternatively, trans-interactions involve integral membrane proteins that can project from each membrane into the cytoplasm. Broadly, tethers are defined as proteins or protein complexes that a) are physically present at the MCS, b) contribute a tethering force between the participating membranes, c) affect the processes taking place at the MCS, and d) modulate the degree of proximity and number of MCSs (Helle et al., 2013; Eisenberg-Bord et al., 2016).

**FIGURE 1:**
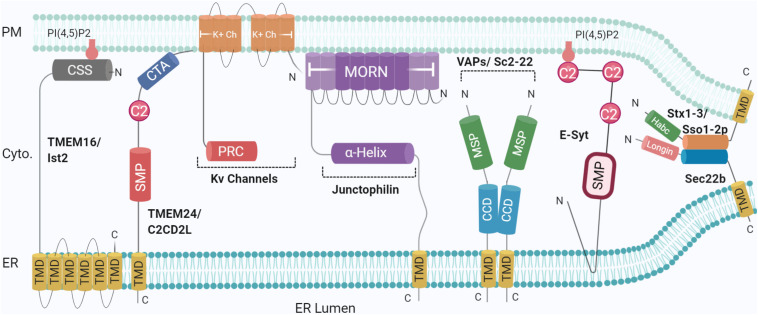
Schematic illustration of the organization and topology of ER-PM tethering proteins. Shown are the so-far identified protein complexes acting in ER to PM membrane recruitment, in yeast and mammals: Sec22b-Syntaxin1/3 (Sso1/2p in yeast) complex, TMEM16 (Ist2 in Yeast), TMEM24 (C2CD2L in Yeast), Kv Channels, Junctophillins, VAPs (Sc2-22 in Yeast), Extended Synaptotagmin (E-Syt). SNARE complexes generate short-range (∼10 nm) MCS whereas other tethers generate longer range (∼20–30 nm). Created with BioRender.com.

Multiple protein families have been indicated at ER-PM junctions, hosting unusual molecular compositions including selected proteomes and potentially lipidomes (Phillips and Voeltz, 2016). No single-family appears to solely perform as a tether. More commonly, tethers are involved in additional functions either by themselves or together with binding partners, identified in model systems and translated to human homologues by sequencing and proteomics. Accordingly, studies presenting ablation of a tethering family generally show reduced numbers of contact sites rather than complete abolition, as exemplified in ER-endolysosomal contacts (Henne et al., 2015) and ER-PM contacts (Manford et al., 2012), further indicating functional redundancy. Loss of all the aforementioned proteins leads to ER-PM dissociation, together with the accumulation of cytoplasmic ER, resulting in misregulation of phosphoinositide (at PM) and Ca^2+^ signalling; compromising cellular integrity, and activating ER unfolded response (Manford et al., 2012; Stefan, 2018; Wang and Dehesh, 2018). The role played by ER-PM MCS in maintaining membrane homoeostasis remains to be investigated. So far, several families of proteins have been identified for their functions in lipid modification and/or transfer between membranes; the TMEM16 family, E-Syt (Extended Synaptotagmins), VAPs (VAMP-associated proteins), Junctophilins, and more recently, SNAREs (Figure 1), as described below.

### Anoctamin/TMEM16 Family

Ist2 is an integral membrane protein predominantly found in the cER in yeast. Studies of the Ist2 family have shown its members can bind to phosphatidylinositol-4,5-bisphosphate (PI(4,5)P_2_) in the PM, via a *C*-terminal basic stretch of 69 amino acids (Ercan et al., 2009; Fischer et al., 2009; Maass et al., 2009). This interaction not only contributes to the localisation of Ist2 to cER but also recruits ER to the PM, creating a compartment devoid of ribosomes (Wolf et al., 2012). Furthermore, deletion of Ist2 increased the distance between ER-PM associations, while overexpression dramatically increased the percentage of cER associated with the yeast PM (Wolf et al., 2012).

The first identified mammalian orthologues, TMEM16A/Ano1 and TMEM16B/Ano2 are Ca^2+^ activated Cl^–^ channels (Yang et al., 2008; Caputo et al., 2008). Other Anoctamins of the 10-member family include TMEM16C (ANO3), TMEM16D (ANO4), TMEM16F (ANO6), TMEM16G (ANO7), and TMEM16J (ANO9) and although some of which can be found localised to ER-PM MCSs (Duran et al., 2012; Bushell et al., 2019), their functions are not fully understood (Picollo et al., 2015). The family members display substantial functional diversity (Milenkovic et al., 2010) and different tissue localisation (Schreiber et al., 2010), though their structural analysis shows a conserved topology including eight *N*-terminal transmembrane domains (TMDs) and stretches of basic amino acids at the *C*-terminus (Pedemonte and Galietta, 2014). As TMEM family members are tethers with an intrinsic activity in lipid transfer (for example, that of phosphatidylserine, i.e., PS) (Lees et al., 2017), it was unexpected to discover that, in yeast, efficient transport of PS to the PM by the cytosolic lipid transfer protein (LTP) Osh6, requires its association with Ist2 (D’Ambrosio et al., 2020). This functionally distinct contribution to PS homoeostasis illustrates the complexity of TMEM16 biology at MCSs. Besides delineating the regulatory role of Ca^2+^ channels, the structural bases of the family members’ functional diversity remain to be elucidated (Picollo et al., 2015). The TMEM16F interactome includes Munc18-1, a partner of the PM SNARE Syntaxin1 (Stx1) (Brooks et al., 2015). TMEM16K, an ER lipid scramblase involved in spinocerebellar ataxia, was found at ER-endosome MCSs further emphasising the importance of this family of proteins. Several ER and endosomal SNAREs were found in close proximity to TMEM16K (Petkovic et al., 2020) but the potential functional relationship is unknown.

### VAPs (VAMP-Associated Proteins)

VAMP-associated proteins or VAPs are another family suggested to act as tethers at MCSs, with orthologues identified in plants (Siao et al., 2016) and yeast (Loewen et al., 2003). VAP’s acronym refer to the initial interaction of VAP-A with the SNARE VAMP in *Aplysia* (Skehel et al., 1995) but the functional relevance of VAP-SNARE interactions is not yet well defined (Weir et al., 2001). VAPs are highly conserved type II integral ER membrane proteins and interact with a wide variety of intracellular proteins (Nishimura et al., 1999; Murphy and Levine, 2016), regulating several cellular processes (Soussan et al., 1999; Kagiwada and Zen, 2003). Structurally, VAPs consist of an *N*-terminal MSP (major sperm protein) domain, a coiled-coil domain involved in dimerisation (Kim et al., 2010) and finally a *C*-terminal TMD, responsible for anchoring in the ER membrane (Murphy and Levine, 2016; Sun and De Camilli, 2018). VAPs are prominently present at several ER-mediated contact sites including with the PM, Golgi, and mitochondria (Eisenberg-Bord et al., 2016). Deletion of the yeast homologues Scs2 and Scs22 result in a loss of cER associated with the PM (Manford et al., 2012). VAPs can also recruit proteins containing an FFAT motif, which consists of two phenylalanine residues flanked by an acidic tract which binds to a positively charged binding motif on the face of the MSP domain (Murphy and Levine, 2016). FFAT motifs have been found in the oxysterol-binding protein (OSBP) related protein family (ORPs), Ceramide transfer protein and *N*-terminal domain-interacting receptor 1–3 (Loewen et al., 2003; Murphy and Levine, 2016). The versatility of this structure gives VAP an opening to interact with multiple proteins, divided into three categories, 1) SNAREs, 2) Viral proteins, and 3) FFAT-motif-containing proteins (Loewen et al., 2003). VAPs, therefore, have a diverse catalogue of binding partners, including LTPs such as oxysterol-binding homology (Osh) proteins and Nir2, located at ER-PM contact sites. Accordingly, the presence of VAP-A and VAP-B is paramount for lipid transport at ER-Golgi contact sites, with knockdown experiments showing detrimental effects on Golgi conformation and distorted composition of phosphatidylinositol-4-phosphate (PI4P), sphingomyelin, and diacylglycerol (DAG) in the Golgi membrane (Peretti et al., 2008).

**FIGURE 2:**
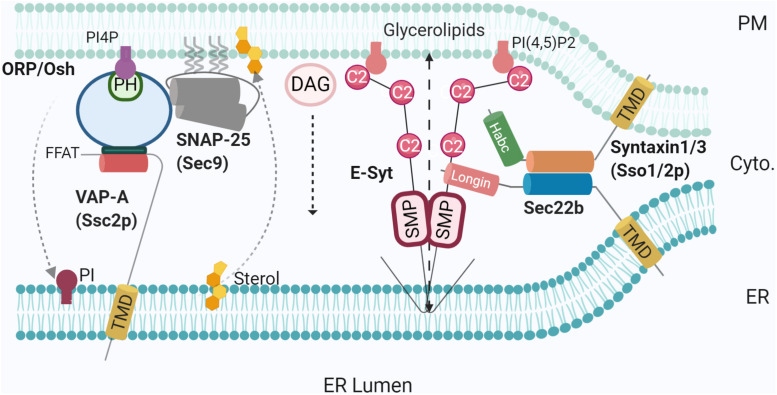
SNAREs and lipid transfer proteins at the MCS. Using varying mechanisms such as FFAT to bridge SNAP25-OSH/ORP-VAP-A, and non-fusogenic Sec22b-Stx1- E-Syt complex, LTPs promote MCS tethering and mediate lipid transfer [PI4P, sterol, glycerolipids, and diacylglycerol (DAG)]. PI4P phosphatase at the ER membrane Sac1 was not represented for simplicity. Created with BioRender.com.

Two groups independently identified an unexpected VAP binding partner in hippocampal neurons. Despite lacking a typical FFAT motif, PM-localised Kv2 channels form ER-PM MCSs via the recruitment of the ER-resident VAPs. This interaction is thought to be regulated by Kv2 phosphorylation (Kirmiz et al., 2018; Johnson et al., 2019), thus involving negative charges acting like the acidic amino acid residues of the FFAT motif of ORPs/Oshs (Sun and De Camilli, 2018) (see below). Incidentally, VAP-A and VAP-B knockdown reduces Kv2 clustering (Kirmiz et al., 2018; Johnson et al., 2019). Finally, VAP-B in particular is associated with the progressive neurodegenerative diseases Amyotrophic Lateral Sclerosis and Parkinson’s disease. Mutations in the MSP domain result in conformational changes that promote oligomerisation and degradation (Kim et al., 2010; Murphy and Levine, 2016), altering ER morphology (Yamanaka et al., 2020). This leads to a range of mechanistic dysfunctions including the different extent of MCS tethering, cytoskeletal coordination, and lipid homoeostasis (Murphy and Levine, 2016) as shown in *Drosophila* (Forrest et al., 2013), highlighting the role of VAPs as an important mediator in ER-PM contact formation (Figures 1, 2).

### JPH (Junctophilins)

Junctophilins were originally identified as junction components in muscle cells, with JPH-2 knockouts presenting reduced associations between the sarcoplasmic reticulum (SR) and PM (Takeshima et al., 2000). While invertebrates have one genomic copy, mammals have four, all identified in excitable cells (Landstrom et al., 2014). Subtypes 1 and 2 are expressed in skeletal and cardiac muscle respectively, while 3 and 4 have been shown to be expressed in hippocampal pyramidal neurons (Nishi et al., 2003). JPHs are embedded in the ER/SR via a C-terminal TMD (Nishi et al., 2000; Takeshima et al., 2000), following a defining feature of the family; eight *N*-terminal MORN (membrane and recognition nexus) motifs, the final two of which are separated by a joining region (Landstrom et al., 2014). These MORN motifs are hypothesised to bind phospholipids, specifically phosphatidylinositol phosphates (PIPs) such as PI4P and PIP_2_ (Landstrom et al., 2014), mediating attachment to the PM according to the number and sequence present, as removal of specific MORN motifs in JPH-1 leads to localisation in the nucleus and cytoplasm rather than PM (Takeshima et al., 2000). There is evidence that the *N*-terminal region (containing the MORN repeats) mediates PM targeting, though the direct involvement of MORN repeats has not been demonstrated (Nakada et al., 2018; Rossi et al., 2019). Between the MORN motifs and *C*-terminal TMD sits an alpha helix, which modulates the distance between the ER/SR and PM, followed by a divergent region of unknown function, so named due to limited sequence conservation between isoforms (Garbino et al., 2009) (Figure 1).

Initially thought to be primarily structural components, Ito et al. (2001) highlights roles for JPHs mediating Ca^2+^ regulation in muscle cells by stabilising a complex of Ryanodine receptor and DHPR Ca^2+^ channels in the SR and PM, respectively. More recently, this has also been observed in T cells (Woo et al., 2016) and several neuronal populations. For example, JPH-3 and -4 double knockouts result in behavioural and physiological defects in mice in addition to dysfunctional Ca^2+^ release and impaired plasticity in hippocampal neurons (Moriguchi et al., 2006). Loss of JPH isoforms prevents the after hyperpolarisation necessary to form Ca^2+^ microdomains (Kakizawa et al., 2008; Takeshima et al., 2015) as JPH-3 and/or -4 are needed to tether and stabilise a Cav1-RyR2-KCa3.1 Tripartite Complex necessary for neuronal excitability regulation (Sahu et al., 2019). Aside from functional studies, JPH-3 has been implicated in neurodegenerative disorders, notably in Huntington’s disease-like (HDL) syndrome. Up to 15% of patients lacking pathological mutations in Huntingtin, the canonical cause of Huntington’s disease, show CAG/CTG repeats in JPH-3 (Holmes et al., 2001) which generate toxic RNA foci and protein aggregation. Loss of JPH-3 in mouse models presents progressive motor abnormalities, consistent with JPH-3 as an, albeit rare, cause of HDL syndrome (Seixas et al., 2012). JPHs have not been connected to SNAREs yet.

### E-Syts (Extended Synaptotagmins)

Extended synaptogamins (E-Syts) are evolutionarily conserved proteins shown to function at ER-PM membrane contact sites in multicellular organisms. Sequence and domain analysis revealed homology with the yeast Tricalbins family, also identified at ER-PM MCSs. The deletion of all three known Tricalbin isoforms in combination with deletion of other associated tethers reduces the number of contact sites (Manford et al., 2012). Tricalbins consist of an *N*-terminal TMD, followed by a synaptotagmin-like mitochondrial lipid-binding protein (SMP) domain and three to five C2 domains (Toulmay and Prinz, 2012), which bind to membranes in a Ca^2+^-dependent manner (Schulz and Creutz, 2004). Indeed, of the three mammalian E-Syt isoforms, E-Syts differs from the yeast orthologues in the number of C2 domains; E-Syt1 has five, while E-Syts2/3 have three C2 domains (Saheki and De Camilli, 2017), enabling recruitment to ER-PM interfaces by membrane-binding of C2 domains upon an increase of cytosolic Ca^2+^ level (Chang et al., 2013; Giordano et al., 2013; Saheki et al., 2016).

In yeast, the Tricalbins are involved in lipid transfer and maintenance of ER curvature, specifically in the formation of cER peaks which are essential for lipid transfer to maintain PM integrity (Collado et al., 2019; Hoffmann et al., 2019). As such, all E-Syts have been shown to perform similar roles under Ca^2+^ regulation. E-Syts2/3 bind PI(4,5)P_2_ at the PM via the C2C domain (Figure 1), but E-Syt1, which is distributed over the whole ER surface area under resting conditions, binds at low Ca^2+^ concentrations via C2E (Giordano et al., 2013). Upon micromolar changes in cytosolic Ca^2+^, E-Syt1 is recruited to ER-PM sites where it can control the distance between the membranes, after Ca^2+^-binding to the C2C domain (Fernández-Busnadiego et al., 2015; Idevall-Hagren et al., 2015). Additionally, hetero- or homodimerisation of SMP domains forms a hydrophobic channel that can facilitate lipid transfer *in vitro* (Schauder et al., 2014; Yu et al., 2016) (Figures 1, 2). Finally, the single E-Syt orthologue in *Drosophila*, catalysing lipid transfer in photoreceptors at the submicrovillar cisternae and the growth of the organism (Nath et al., 2020), was also suggested to modulate synaptogenesis (Kikuma et al., 2017). E-Syts have since been identified in mammalian neuronal cells but, while it is plausible that they can perform these functions (Giordano et al., 2013; Fernández-Busnadiego et al., 2015) their physiological significance in neurons is so far unclear and may only be noticeable under specific conditions. Indeed, hippocampal neurons from E-Syt triple knockout mice exhibited no change in ER morphology or protein composition, and neuronal survival under stress was not impaired, suggesting that other tethers may provide compensation for their loss (Sclip et al., 2016). ESyts have recently been connected to SNAREs as discussed below.

### SNAREs

Soluble *N*-ethylmaleimide-sensitive factor attachment protein receptors (SNAREs) constitute the basic molecular machinery of intracellular membrane fusion. Positioned on opposite membranes, vesicular SNAREs on one side and target SNAREs on the other assemble in a ternary complex which allows for close docking and subsequent fusion as exemplified in the case of synaptic vesicle fusion with the presynaptic PM (Galli and Haucke, 2004). ER-localised vesicular SNARE Sec22b contains an *N*-terminal Longin domain, followed by the characteristic SNARE domain and finally a C-terminal TMD (Petkovic et al., 2014) (Figure 1). Previously shown to be involved in trafficking between the ER and Golgi via its interaction with t-SNAREs Syntaxin 5, membrin, and rbet1 (Williams et al., 2004), Sec22b was shown to associate with Stx1 at ER-PM contact sites in the growth cones of developing neurons (Petkovic et al., 2014). This complex does not include SNAP23/25/29/47. However, liposome assays, while confirming a fusogenic activity of Sec22b in a tripartite assembly with Stx1 and SNAP-25, did not elicit the fusion of membranes in the absence of SNAP25. Additionally, Sec22b was not found at the cell surface. Altogether, this evidence suggest that Sec22b/Stx1 association does not lead to membrane fusion. This assembly represents a new short range (∼10 nm) tether between ER and the PM (Petkovic et al., 2014) able to determine MCS distance, as elongating the linker between the SNARE and TMD domains of Sec22b with polyproline motifs increased the distance between the ER and PM.

Meanwhile, Sec22b was also essential for neuronal development, as RNAi-mediated knockdown of Sec22b inhibited axonal and dendritic growth. Altogether this supports the notion that the complex is not involved in membrane fusion but instead acts as a tether and facilitates additional functions such as lipid transfer. Indeed, in the brain, E-Syts were recently found in complexes containing Stx1 or Stx3 and Sec22b but not SNAP25 (Gallo et al., 2020). Notably, the interaction between the ER-resident ESyts and Sec22b depends on the latter’s Longin domain. Removal of the Longin domain induces recovery of SNAP25 in Sec22b immunoprecipitates. Therefore, Sec22b Longin domain likely excludes SNAP25 allowing the occurrence of a non-fusogenic Sec22b-Stx1 complex. Super-resolution imaging of growth cones in developing neurons demonstrated the very close proximity of E-Syt2 and Sec22b beneath the PM, plausibly populating ER-PM contact sites. Moreover, the interaction between E-Syt and Sec22b seems to promote and/or stabilize the association of Sec22b with Stx1/3. E-Syt overexpression is responsible for neuronal membrane expansion in the form of filopodia leading to protuberant hyper-ramified axons, an effect which depends on Stx1 and Sec22b as shown using clostridial toxins and expression of Sec22b extended by a polyproline spacer and the Longin domain. Brought together, these observations allow postulating E-Syt engagement in a tripartite complex with Sec22b and Stx1/3, regulating lipid transfer to the PM within ER-PM contact sites (Gallo et al., 2020). This situation provides the first example of a SNARE-containing MCS where an aborted formation of a fusogenic SNARE complex contributes to the function of an LTP, namely E-Syts. It further emphasises the importance of Longin domains conserved molecular functions in SNARE regulation via promoting and inhibiting membrane fusion by folding back onto the fusion-inducing SNARE coiled-coil domain, along with managing Longin SNAREs interactions with proteins controlling intracellular sorting (Daste et al., 2015; Gallo et al., 2020; Singh et al., 2020).

### Other ER-PM Tethers

In addition to the above tethering proteins, which appear to be ubiquitously expressed among mammalian cells, there is recent evidence for tethering candidates exclusive to neuronal populations. For example, TMEM24/C2CD2L is an ER-localised protein shown to transport phosphatidylinositol between the ER and the PM via its SMP domain, binding to the PM in a Ca^2+^-dependent manner (Lees et al., 2017). TMEM24 is also enriched at ER-PM MCSs in neurons and acts as a tether. TMEM24 is redistributed throughout the ER at high cytosolic Ca^2+^ levels and may interact with other potential tethers including VAPs and Kv2 channels, indicating a regulatory loop related to neuronal activity (Sun et al., 2019) (Table 1).

**TABLE 1:** Summary of the structural and functional actors at ER-PM membrane contact sites.

Protein	Structure	Cell type	Organelle	Binding pattern	Interactions	References
TMEM16	Evolutionary conserved eight TMDs and C-terminal stretch of basic amino acids	Yeast – Mammal	Cortical ER	Contacts PM via highly basic COOH terminus	IP3R1, Osh6, Munc18-1	Pedemonte and Galietta, 2014; Brooks et al., 2015
VAMP-associated proteins (VAPs)	N-terminal MSP domain, a coiled-coil domain and a C-terminal TMD	Yeast and plant	ER-mediated contact sites including with the PM, Golgi, and mitochondria	Binds via MSP domain to a FFAT motif located on the binding partner	Osh, Nir2, Kv2	Murphy and Levine, 2016; Johnson et al., 2019
Junctophilins (JPH)	Eight N-terminal MORN motifs with C-terminal TMD sits and alpha helix between them	Mammal – Muscle (skeletal and cardiac) + Pyramidal neurons	ER residents	Binds PM via MORN domains	PIPs – PI4P and PIP_2_	Nishi et al., 2000; Landstrom et al., 2014
Extended synaptotagmins (E-Syt)	SMP-domain-containing tethers with N-terminal TMD and three to five C2 domains	Yeast, and mammal	ER residents	Ca^2 +^ dependent dynamic tether. E-Syt1 binds at low Ca^2 +^ concentrations via C2E E-Syts2/3 bind PI(4,5)P2 at the PM via the C2C domain	Sec22b-Stx1; SNAP25	Giordano et al., 2013; Gallo et al., 2020
SNARE Sec22b	N-terminal Longin domain, SNARE domain and a C-terminal TMD	Yeast and mammal	ER-localised	Hypothesized to recruit E-Syt via its Longin domain, involved in non-fusogenic Sec22b-Stx1 complex.	Stx1/3, SNAP-25, E-Syt	Petkovic et al., 2014; Gallo et al., 2020
Kv2 channels	C-terminus with proximal restriction and clustering (PRC) domain	Mammal (Brain), plants	PM -localised	Clusters opposite to astrocytic processes, mediating intercellular communication with non-neuronal cells, in addition to regulating synaptic firing	Ryanodine receptor, VAPs, Stx1, SNAP-25	Du et al., 1998; Fili et al., 2001; Leung et al., 2003; Johnson et al., 2019
TMEM24	Anchored to the ER membrane via an N-terminal transmembrane span, followed by the SMP domain, a C2 domain, and C-terminal region (CTR)	Mammal- neuronal property i.e., pancreatic β-cells, fibroblasts	ER-localised	Binding to the PM in a Ca^2+^-dependent manner	VAPs and Kv2 channels	Sun et al., 2019
Oxysterol-binding protein (OSBP) related proteins (ORPs)	FFAT motif or C-terminal TMD and membrane-binding domains such as PH domain	Yeast – Mammal	Anchored to PM tethering to ER	Binding to the ER transmembrane protein via the FFAT motif	VAP, Scs2p, Sec9p	Lehto et al., 2001
Steroidogenic acute regulatory protein-related lipid transfer (StARt)	210 conserved amino acid sequence, folding into an α/β helix-grip structure with hydrophobic binding pocket	Yeast – Mammal	Anchored to ER, tethering to PM	Bridging is enabled through phospholipid – GRAM domain and shuttle sterols present through START-like domain	Binds with Cholesterol and interacts with VAP	Besprozvannaya et al., 2018; Sandhu et al., 2018; Naito et al., 2019

Widely expressed throughout mammalian brain Kv2 K+ channels are distinguished for their neuronal surface localisation pattern divided into a) freely diffusive channels in PM and b) micron-sized clusters present on the soma, dendrites, and axon initial segment (Johnson et al., 2019). Kv2 voltage-gated K^+^ channels are frequently identified as interactor partners with known tethers. However, several lines of evidence have recently implicated Kv2 channels as tethers in their own right. Multiple studies identified Kv2 (Kv2.1 and Kv2.2) channel clustering at the PM in hippocampal neurons (Antonucci et al., 2001), cerebellar Purkinje cells (Kaufmann et al., 2009), and even Aplysia neurons (Zhang et al., 2008) due to a 26-amino acid long targeting sequence called the Proximal Restriction and Clustering domain (Lim et al., 2000). While early studies recognised clusters at close appositions, more recent studies have shown that Kv2 clustering remodels the ER, initiating the formation of ER-PM contact sites (Fox et al., 2015; Kirmiz et al., 2018), where L-type Ca^2+^ channels and Ryanodine receptors are recruited to mediate burst firing (Mandikian et al., 2014; Irie and Trussell, 2017; Kirmiz et al., 2018). Further investigation conducted by Kirmiz et al. (2019) has shown that Kv2.1 knockout mice have significantly reduced PI4P and PI(4,5)P2, suggesting their integral involvement in PI4P and PI(4,5)P2 regulation; components crucial for generating secondary messengers inositol trisphosphate (IP3) and DAG (Balla et al., 2020; Jing et al., 2020). However, clustering at close appositions was not equal in all cells; Kv2.1 (also called BK channels) expression differed between pyramidal neurons and interneurons of the hippocampus, potentially in reference to their different functions (Antonucci et al., 2001). Furthermore, Kv2.1 channels in the soma are commonly found clustering opposite to astrocytic processes, so may mediate a method of intercellular communication with non-neuronal cells, not just by regulating synaptic firing (Du et al., 1998).

Interestingly, disrupting the phosphorylation-dependent interaction between Kv2.1 and VAP-A (Kirmiz et al., 2019) is neuroprotective and prevents pro-apoptotic K^+^ entry upon ischaemic injury (Schulien et al., 2020). It is particularly interesting that Kv1.1 and Kv2.1 channels were also found to interact with Stx1 (Fili et al., 2001) and SNAP-25 (MacDonald et al., 2002; Leung et al., 2003). These interactions appear conserved in plants where they were shown to regulate membrane expansion for cell growth independently of vesicle traffic (Honsbein et al., 2009). Future studies should address the potential role of Kv-SNARE interactions in regulating MCSs’ protein complexes and lipid transfer.

### Functions Mediated at ER-PM MCSs

The characterisation of the molecular composition of MCSs has aided the evaluation of their physiological significance, with most tethering proteins seemingly mediating functions such as Ca^2+^ homoeostasis or lipid transport. We will thereafter go on to review the recognised functions occurring at MCSs and their significance in neurons.

### Lipid Transfer at ER-PM MCSs

The translocation of lipids between membranes has been shown since some of the earliest studies (Vance, 1990). More recent evidence has begun to shed light on the potential mechanisms and the components involved. LTPs are able to bind lipids in one membrane and deliver them to another, closely situated membrane. Consequently, it is logical that LTPs function at MCSs (Toulmay and Prinz, 2012). While the mechanisms of translocation are still contested, there are two prevailing theories related to their mechanism of action. The tunnel model proposes that lipid-binding domains form a tunnel bridging the intermembrane space, with a small channel open along the length of the domain, such that the polar head group is exposed to the hydrophilic environment as it traverses the channel (Schauder et al., 2014). Hydrophobic residues line the inside of the tunnel stabilising the tails, with conformational changes revealing lipid-binding sites at the tunnel entrance, permitting lipid binding and translocation to the opposing membrane (Schauder et al., 2014; Lees and Reinisch, 2020). Alternatively, the shuttle model suggests that LTPs specifically deliver lipids from the donor membrane, particularly across short distances (under ∼200Å). A series of conformational changes extract the lipid from the single entry point along the closed hydrophobic environment and insert the lipid into the acceptor membrane (Schauder et al., 2014; Wong et al., 2019). Some LTPs have been shown to dimerise to form the tunnel, while others utilise β-barrels (Im et al., 2005), α-helices (Tong et al., 2018), or a mix to form the bridge (Wong et al., 2019). In concert with this, it has been suggested some LTPs (e.g., E-Syt2) may necessarily interact with partners, including other LTPs, to discriminate and transport specific lipids (Schauder et al., 2014). The channel model of E-Syts action clearly fits well with the notion that lipid transfer would work best at the closest contacts such as those involving SNAREs (<10 nm) (Gallo et al., 2020) (Figure 2).

**FIGURE 3:**
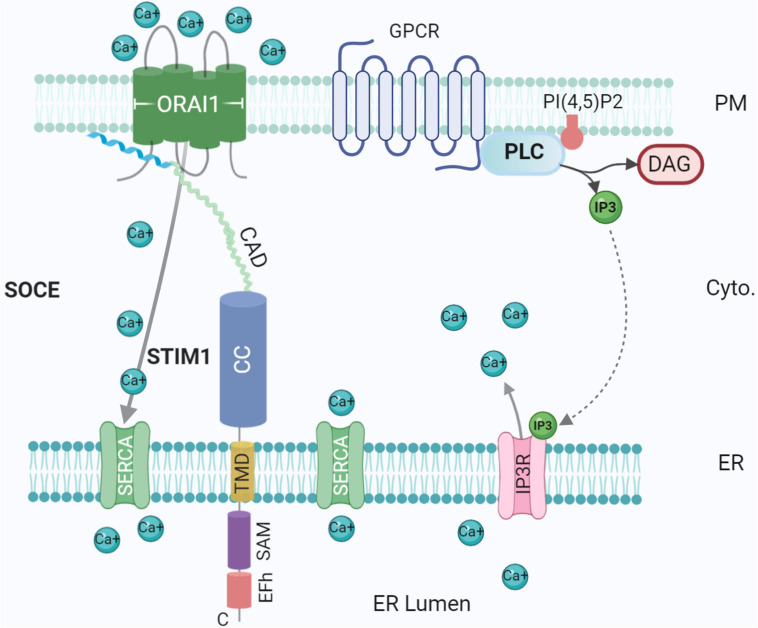
Ca^2+^ influx at the ER-PM contact site. Tethering proteins involved in the control of the Ca^2+^ ions fluxes between the ER lumen, the cytosol and the extracellular medium: ORA1, STIM1, SERCA, and IP3 Receptor (IP3R). IP3R is activated by IP3 generated by Phospholipase C (PLC) upon activation of G-protein coupled receptor (GPCR). The G protein subunits ware not represented for simplicity. Created with BioRender.com.

Sequence analysis of SMP domains, identified in proteins of the ERMES complex in yeast ER-mitochondrial MCSs (Lee and Hong, 2006), showed homology with the TULIP superfamily, confirming lipid-binding interactions (Kopec et al., 2010; Alva and Lupas, 2016). SMP domains have been shown to dimerise in order to facilitate lipid transport. A shuttle mechanism, as described above, likely operates in E-Syt as the 90Å-long SMP dimer is too short to span the distance between membranes (Schauder et al., 2014). E-Syts were found to mediate the transport of glycerophospholipids in a bidirectional manner using *in vitro* assays (Saheki et al., 2016; Yu et al., 2016; Bian et al., 2018; Bian and De Camilli, 2019). E-Syts might also participate in mechanisms altering lipid composition. For example, PI(4,5)P_2_ hydrolysis following phospholipase C (PLC) activation by mediated via G-protein coupled receptor (GPCR) (Figure 3) led to the release and accumulation of DAG in E-Syt knockout cells (Saheki et al., 2016). In addition, Ca^2+^ influx is needed to relieve autoinhibition of the SMP domain in E-Syt1 by the C2A domain (Yu et al., 2016; Bian et al., 2018). Furthermore, a study in human embryonic kidney cells and rat superior cervical ganglia neurons has shown that E-Syt2 stabilises Sac1, an ER-localised lipid phosphatase, at ER-PM MCSs (Dickson et al., 2016). Here, Sac1 dephosphorylates phosphatidylinositol monophosphates; overexpression of Sac1 increased PI levels approximately two-fold, while PI4P and PIP_2_ levels decreased. In accordance with this role, Sac1 and E-Syt2 were shown to co-localise while E-Syt2 knockdown significantly reduced Sac1 puncta at MCSs coinciding with accumulation of PI4P and PIP_2_ (Dickson et al., 2016). A role of E-Syt2 in PM PI regulation fits well with the observation that E-Syts regulates growth as shown in *Drosophila* (Nath et al., 2020) and mammalian neurons (Gallo et al., 2020) because of the role of PIP_2_ in PM dynamics (Scholze et al., 2018). Furthermore, E-Syts were found to interact with FGFR1 (Tremblay et al., 2015), a receptor that regulates PM PIP_2_ (Herdman and Moss, 2016).

Another major class of LTPs are ORPs (Table 1). There are 12 members of the ORP family in humans and seven in yeast (homologue Osh proteins), each containing a conserved OSBP-related domain (ORD). In addition, members usually contain ER-targeting sequences (e.g., FFAT motif or *C*-terminal TMD) and membrane-binding domains, most commonly a Pleckstrin homology (PH) domain (Lehto et al., 2001). Recently, studies have shown that ORPs facilitate lipid transport at several MCSs, including ER-lipid droplets (Du et al., 2020) and ER-PM MCSs (Chung et al., 2015; Mochizuki et al., 2018). The function of ORPs (and Oshs) relies on their binding to the ER transmembrane protein, VAP (Scs2p in yeast) via the FFAT motif (Loewen et al., 2003). In yeast, Osh2/3p-Scs2p complexes are located at ER-PM MCSs (Weber-Boyvat et al., 2015) where they are thought to be responsible for the extensive tethering of the cortical ER and PM (Figure 2). Although being topologically adapted to this function through selective Scs2p binding, Osh were also shown to be highly integrated in an intriguing interplay with SNARE proteins (Weber-Boyvat et al., 2020). This later study demonstrated the direct interaction of Osh and the SNARE domain of Sec9p (SNAP25 homologue), along with the previously defined Sec9p interaction with Sso1 (Stx homologue). Furthermore, this crosstalk was conserved in hippocampal neurons. Knocking down ORP2 in neurons reduced both neurite growth and synaptogenesis, further emphasising the importance of lipid transfer. In this situation, a plausible hypothesis is the recruitment of Sec9p (SNAP25) by an Osh-Scs2p complex reinforcing tethering between ER and PM (Figure 2). In the absence of an ER-resident v-SNARE and thus membrane fusion, negative regulation of exocytosis by Osh overexpression (Weber-Boyvat et al., 2020) could be due to a re-routing of Sec9p (SNAP25) by Osh towards a ternary Osh-Scs2p-Sec9p complex, allowing only lipid transfer/modification.

Additional sterol transporters were identified as StARt (steroidogenic acute regulatory protein-related lipid transfer) domain (StARD) proteins (Table 1), also known as the StARkin superfamily (Jentsch et al., 2018). Following, LAM/Ltc proteins were identified in yeast to contain StART-like domains, structurally similar to the StART domains (Gatta et al., 2015). LAM/Ltcs are localised to the ER via a C-terminal TMD and can be found at ER-PM MCSs. A hydrophobic cavity the StARt domain accommodates sterols, supported by slowed sterol transport upon deletion (Horenkamp et al., 2018; Tong et al., 2018). LAM isoforms also contain a PH-like domain, *N*-terminal to the StARt domain, thought to be involved in targeting, although unable to bind lipids (Jentsch et al., 2018; Tong et al., 2018). In mammals, several StART (e.g., StARDs) and StART-like domain-containing proteins have been suggested to transport cholesterol to the PM and between intracellular organelles (Horenkamp et al., 2018; Clark, 2020). For example, mammalian homologues of yeast LAM proteins GRAMD1s/ASTERs contain a C-terminal TMD localising the protein at the ER, an *N*-terminal GRAM domain associating with the PM and a StART-like domain (also called VaST domain) to facilitate lipid transfer (Sandhu et al., 2018; Naito et al., 2019). While consistent with a role in sterol homoeostasis at MCSs, the potential physiological function in neurons remains to be investigated.

While much of the mechanistic detail describing lipid transfer remains elusive, the diversity among the identified molecular components (and potential for unidentified members) indicates extensive functional redundancy, highlighting the significance of lipid homoeostasis in biological membranes. Enhancement of ER-PM MCSs by E-Syt recruitment also induces translocation of Nir2 to ER-PM MCSs, potentially via interactions with VAP isoforms (Amarilio et al., 2005). Here, Nir2, a phosphatidylinositol transfer protein, replenishes PIP_2_ in the PM by promoting transfer from the ER, offering a feedback loop for receptor-induced Ca^2+^ signalling (Chang et al., 2013). Abnormal localisation and disproportionate lipid concentrations can have dire effects on organelle function and downstream signalling pathways. In this manner, it has been suggested LTPs do not simply move lipids between compartments, but they also present them to metabolising enzymes to maintain specific compositions (e.g., Sac1 and E-Syt2 localisation). For example, studies in yeast show that the lipid transporter Osh3 is needed not only for PI4P transport, but is independently necessary for the localisation of Opi3, a phosphatidylethanolamine *N*-methyltransferase involved in phosphatidylcholine synthesis (Tavassoli et al., 2013). In accordance with roles in signalling pathways, temperature stress in yeast induces recruitment of phosphoinositide-dependent kinase orthologues by PI4P and PIP_2_, modulating the PDK-TORC2-Akt cascade thus activating sphingolipid synthesis in the ER. A key component of the PM, sphingolipid transfer from the ER is essential in maintaining PM integrity under stress (Omnus et al., 2016). Following, the same study showed that deletion of ER-PM tethers resulting in dysfunctional Ca^2+^ signalling and subsequent impaired sphingolipid synthesis, manifesting in PM integrity defects.

## Ca^2+^ Homoeostasis and Signalling

Ca^2+^ is an essential second messenger utilised ubiquitously in eukaryotes. Early work by Michell (1975) suggested an integral role played by ER-PM contacts in Ca^2+^ signaling. A prerequisite for Ca^2+^ signaling is a low baseline Ca^2+^ concentration in the cytosol; in resting cells, Ca^2+^ levels are actively maintained in the nanomolar range, several orders of magnitude below typical extracellular concentrations (Carrasco and Meyer, 2011). With low intracellular concentrations, Ca^2+^ is acquired from the extracellular milieu and/or from specific stores in ER, Golgi, and mitochondria (Giorgi et al., 2018). When low, these stocks must be replenished from the extracellular milieu, requiring signalling between organelles, thus MCSs are obvious candidate hubs for signal transduction (Wu et al., 2006). Post ER Ca^2+^ depletion, mediated by GPCR, PLC and IP3, Ca^2+^ is replenished through a process called store-operated Ca^2+^ entry (SOCE) (Figure 3), directly mediated by ER-PM junctions through ER Ca^2+^ sensor STIM1 and PM Ca^2+^ channel Orai1 (Michell, 1975; Chang et al., 2013). These form an elementary unit upon Ca^2+^ depletion, sensed by the lumenal EF domain of STIM1 protein, generating cytoplasmic Ca^2+^ signals and refilling ER Ca^2+^ (Balla et al., 2020; Stefan, 2020) (Figure 3). Defects in this mechanism could contribute to degenerative diseases such as Alzheimer’s disease (Popugaeva and Bezprozvanny, 2014).

At rest, STIM1 and Orai1 are evenly distributed across ER/PM, however, post ER Ca^2+^ depletion, STIM1 is activated and releases Ca^2+^ from the lumenal EF-hand, eventually enabling oligomerization and translocation. STIM1 in activated state directly interacts and gates Orai1 Ca^2+^ channel (Jing et al., 2020). Orai1 permits the entry of Ca^2+^ into the cytosol, where repletion of the ER as a Ca^2+^ store is facilitated by SERCA channels (Luik et al., 2008; Chang and Liou, 2016). Mammals express two STIM isoforms, which share high sequence homology (Berna-Erro et al., 2009). Both isoforms are widely expressed in the brain, although show differential patterns with STIM1 more common in Purkinje neurons of the cerebellar cortex (Hartmann et al., 2014) while STIM2 is the predominant isoform expressed in the hippocampus (Berna-Erro et al., 2009) and cortex (Gruszczynska-Biegala et al., 2011). Further research has revealed additional regulatory and stabilising partners. Receptor-induced PIP_2_ hydrolysis results in Ca^2+^ influx due to the generation of IP3 and subsequent opening of IP3 receptors, Ca^2+^ channels in the ER. Additionally, E-Syts are Ca^2+^-activated, transferring PLC generated DAG from the PM to ER (Saheki et al., 2016). Recruitment of E-Syt1 to ER-PM junctions (Chang et al., 2013; Idevall-Hagren et al., 2015) modifies ER which form a ring around the site of Ca^2+^ entry, thereby stabilising MCSs and accelerating Ca^2+^ replenishment (Kang et al., 2019). Furthermore, localisation at PI(4,5)P_2_-rich microdomains tethered by E-Syt1 seems to be necessary for binding and inactivation of STIM by SARAF (Maléth et al., 2014). Interestingly, knockdown of Sec22b does not impair SOCE but increasing the length of Sec22b strongly affects SOCE (Petkovic et al., 2014), suggesting that different tethering mechanisms could operate in an independent manner at ER-PM MCSs to a limited extent. In addition, an essential role of α-SNAP, a SNARE interactor, was found in binding to and mediating functional coupling of Stim1 and Orai1. This function for α-SNAP is direct and independent of its known activity in NSF dependent SNARE complex disassembly (Miao et al., 2013).

Another intriguing protein family playing a critical role in modulating Ca^2+^ at ER-PM MCSs is GRAMD1/Aster. GRAMD1 and GRAMD2 localise to separate MCSs, suggesting different physiological functions. For example, GRAMD2 localisation at ER-PM MCSs is PI(4,5)P_2_-dependent, highlighting the MCS for recruitment and translocation of STIM1 post depletion of Ca^2+^ (Besprozvannaya et al., 2018).

## Rising Matter: ER-PM MCSs and Autophagy

Lipid transfer is essential to maintain cell homoeostasis and simultaneously mediate dynamic changes upon stimulus. Indeed, transfer can modulate lipid synthesis, cell growth and is also vital for organelle biogenesis and function, as each membrane possesses specific lipid compositions, including asymmetric distributions between leaflets. This requires targeted and often large-scale transport of lipids throughout the cell (Prinz, 2014). Lipid transfer operates complementarily to vesicular transport and membrane fusion. Contrary to the latter, lipid transfer does not imply full lipid mixing between organelles, nor co-transfer of proteins and can operate for amounts of molecules not defined by quantal size of organelles. This complementarity between vesicular transport and lipid transfer at contact sites is illustrated by the formation of the phagophore, the initial step of autophagy. For instance, macroautophagy is dependent on the formation of the phosphatidylinositol-3,4,5-trisphosphate (PIP_3_)-enriched phagophore, which requires progressive vesicular fusion to form the maturing structure (Rubinsztein et al., 2012; Wang et al., 2016) and also lipid transfer by ATG2 (Maeda et al., 2019; Sawa-Makarska et al., 2020). Further evidence suggests that ER-PM contact sites can be directly involved in autophagy. Induction of autophagy by nutrient deprivation results in upregulated and stabilised E-Syt-mediated MCSs in mammalian cells. Co-localisation of phagophore markers such as LC3 and DCFP1 with E-Syt2 or 3 suggests that about 30% of all autophagosome assembly occurs at ER-PM MCSs (Nascimbeni et al., 2017). SiRNA-mediated knockdown of E-Syt2 and 3 resulted in decreased expression of some members of the PI3KC3 complex, such as Beclin1 and VMP1. Both precipitated with E-Syt2, suggesting E-Syts stabilise the PI3KC3 complex, needed for local PI3P synthesis, therefore making E-Syt-mediated ER-PM MCSs ideal locations for autophagosome assembly. The extent of this mechanism in neurons and how this may contribute to a disease characterised by deficiencies in the autophagy-lysosomal pathway has yet to be investigated. Sec22b was suggested to mediate the fusion of autophagosome with the PM via interaction with syntaxin 3/4 and SNAP-23/29 in on neuronal cells (Kimura et al., 2017). It is tempting to speculate that autophagosome Sec22b might differ from ER Sec22b in a regulatory process, maybe related to the Longin domain, which would allow for fusion with the PM in the case of the autophagosome but not the ER.

## Conclusion

The study of MCSs has grown tremendously in the last 20 years, however, our understanding of their structure and functions still shows many gaps. Much of this difficulty arises from the inherent features of MCSs; they are minute, dynamic structures with diverse molecular compositions between organelles and organisms and thus can be difficult to study. ER-PM MCSs, while relatively well characterised, exemplify the complexity surrounding MCSs, with multiple tethering complexes and binding partners defining the structure’s performance as a signalling hub and as key homeostatic regulators, responsive to physiological and stressful conditions. What remains a major question in the field is the relationship between MCS and particularly their lipid transfer activity with membrane fusion events. The fact that SNAREs, which constitute the basic membrane fusion machinery, would interact with LTPs and be found both at MCSs suggest that their regulation might be key to either generate a contact or fuse. In such scenario, we propose an important function for the Longin domain of Sec22b. In other words, could membrane contact correspond to a membrane docking with frustrated fusion? An evolutionary view of these processes would certainly solve this question.

## Author Contributions

BH wrote initial version and revised last version. NS and CV extensively rewrote and completed. NS designed the figures using BioRender. TG supervised and wrote final draft.

## Conflict of Interest

The authors declare that the research was conducted in the absence of any commercial or financial relationships that could be construed as a potential conflict of interest.
